# The Role of Parental Acceptance–Rejection in Emotional Instability During Adolescence

**DOI:** 10.3390/ijerph16071194

**Published:** 2019-04-03

**Authors:** Santiago Mendo-Lázaro, Benito León-del-Barco, María-Isabel Polo-del-Río, Rocío Yuste-Tosina, Víctor-María López-Ramos

**Affiliations:** 1 Department of Psychology, Faculty of Teacher Training College, University of Extremadura, Caceres 10071, Spain; mabelpdrio@unex.es (M.-I.P.-d.-R.); vmlopez@unex.es (V.-M.L.-R.); 2 Department of Educational Sciences, Faculty of Teacher Training College, University of Extremadura, Caceres 10071, Spain; rocioyuste@unex.es

**Keywords:** emotional instability, adolescence, parental affect, parental rejection

## Abstract

The present study focuses on analyzing the relationship between the parental acceptance–rejection perceived by adolescents and emotional instability from the early stages of adolescence. Special attention will be paid to potential differences between mothers and fathers. A total of 1181 students, aged 11–17, took part in the study. We used the factor of emotional instability in the Big Five Questionnaire (BFQ-NA) and an affect scale (EA-H) to measure parental acceptance–rejection. The analyses performed show a clear association between emotional instability with maternal/paternal criticism and rejection. Specifically, maternal criticism and rejection in early adolescence and paternal criticism and rejection in middle adolescence were associated with emotional instability, confirming the association between children’s and adolescents’ emotional adjustment and family dynamics. This study makes interesting contributions to understanding paternal and maternal rejection during the different stages of adolescence. These differences should be incorporated into the research on parental influence and its role in the development of personality among adolescents.

## 1. Introduction

Parental influence and its crucial role in child development is widely acknowledged. Understanding the individual differences in the relationships between parents and adolescents is key, because the quality of these relationships shapes the development of adolescents [1]. Different research has focused on personal and social variables that are relevant for children and the way in which parental behavior, upbringing styles, or educational styles are associated with personality [2,3,4,5,6].

The process of shaping personality is the result of a person’s temperament, the upbringing they receive from those around them (especially their parents), and the relationships they establish with these people. In the 1980s it was argued that all the personality traits could be accounted for by five major factors that comprise all the variations on people’s conduct and manifest behavior [7]. McCrae and Costa’s Big Five Model [8] is one of the most widely accepted paradigms for the study of personality, and it constitutes a valuable representation of the structure of personality in children and adolescents [9,10]. This model puts forward five dimensions, enabling the identification of adolescents in terms of social adjustment and adaptation [11].

Just like the effect of children’s upbringing influences their development, their personality, and their socioemotional adjustment [4], their manifest behavior may have an effect on the way they interact with their parents. Palacios [12], in his multiple influence model, stressed the fact that parent–child relationships are bidirectional. Later research also indicates that these interactions involve an influence that is mutual, bidirectional, and circular [13]. Thus, some studies suggest more specifically that there is a mutual, bidirectional link between the emotional adjustment of children and adolescents and family dynamics [14,15]. Parents are very sensitive to their child’s temperament; they do not simply tell them what has to be done, but they adapt their demands and their upbringing styles to the child’s temperament [16]. This parental conduct, in turn, shapes the future behavior of their children [17].

Temperamental differences are often established by virtue of several factors. Among these, we find emotional response [18]. Indeed, emotions in children and adolescents somewhat mediate the quality of parents’ positive expressiveness toward their offspring [19]. Some authors [20] point out that parental socialization correlates with negative emotionality during infancy. In this regard, emotional instability [8] reveals itself as a factor that contributes towards people’s vulnerability and poor adaptation, their negative view of events, and exteriorization of emotional overreaction, together with certain lack of self-control [18]. This factor may be heightened in children and adolescents as a result of some parental upbringing styles [21].

Parental upbringing styles are the result of the interaction of parents’ attitudes and behaviors when they interact with their children [22], leaving a direct imprint on their conduct as a result. Thus, they facilitate the development of their personality and they provide emotional security and wellbeing [23,24,25]. The different parenting styles (authoritative, permissive, authoritarian, and neglectful) pivot around two major dimensions: affect/communication on the one hand, and control/demandingness [22,26,27]. The dimension of parental affect/communication refers to the extent to which parents show their care for and acceptance of their children [23]. In this regard, Rohner [28] identifies two characteristics of parental conduct: acceptance and rejection—a continuum in which expression may range from love and affection by parents who accept their child to aversion or reprobation by parents who show rejection.

Parental acceptance–rejection theory [29,30] is based on evidence that indicates that its main dimension (i.e., the acceptance–rejection axis) shapes certain aspects of parental behavior that are developed during the upbringing process. Parental acceptance is associated with greater psychological adjustment, whereas parental rejection is associated with psychological disorders [31]. Some studies conclude that the psychological and social adjustment of children and adolescents is different depending on whether the relationships with their parents are based on acceptance or rejection [15,29].

If we take these dimensions for granted, when parent–child relationships are characterized by affection and communication in the family environment and by flexibility in the establishment and adherence to rules, then family conflicts help to transform these relationships and facilitate the development of adolescents. By contrast, the lack of affection and communication, strict norms, and a negative view of children—and the conflicts thereof—may bring about negative effects in the personal and socio-emotional development of adolescents [32]. Parents’ positive affect toward their children is regarded as a protective factor against mental health disorders in children [33,34], whereas those children who experience rejection from their parents have a higher prevalence of internalizing mental disorders (passivity, apathy, feelings of despair and depression, and nervous alterations, among others), which are typical of certain personality traits, more specifically emotional instability [35,36,37,38,39,40,41]. 

Some research has tried to analyze the role of maternal/paternal acceptance–rejection separately on the psychological adjustment of children. Their conclusions suggest that both types of acceptance–rejection are equally important in all cultures, and that it is associated in similar terms to children of both sexes [42]. However, others find that children’s psychological adjustment can be better accounted for through maternal acceptance–rejection [43] or paternal acceptance–rejection [44,45]. In this regard, the best predictor of mental health disorders (whether internalizing or externalizing) is not so much the gender of the parents, but the relationships between them and their children [46,47,48]. 

Children, in these interactions with their parents, tend to assess their mothers as having a higher degree of affect, control, and discipline strategies when compared to their fathers. With regards to hostile behavior and rejection, fathers and mothers are perceived roughly on equal terms [49]. By contrast, some research claims that it is mothers who rank higher on criticism–rejection [50] while others draw similar conclusions for fathers [51].

Yet, there are differences in this scale depending on the gender of the children. Thus, females perceive higher parental acceptance. In addition, the sole presence of the father, regardless of the perceived paternal acceptance, affects psychological adjustment [52]. Meanwhile, males perceive the highest paternal rejection [53,54,55]. These results support the popular belief that parents are still more rigid, hostile, controlling, and critical of their male children [49].

The age of the children also seems to point towards different results. The youngest children perceive higher involvement and supervision by both parents, and the elder children perceive a higher degree of hostility and negligence in parental behavior, especially in the case of their mothers [49]. Participation and parental control are robust predictors of achievement in adolescents [56]. During this stage, fathers and mothers display a greater amount of rejection behaviors, hostility, and permissiveness toward children [57]. 

Within the interpersonal acceptance–rejection theory (IPARTheory) [58], the personality sub-theory postulates the universality of the response of male and female children to perceived parental acceptance–rejection, regardless of their social, cultural, racial, or ethnic context. Thus, this theory attempts to explain the consequences that children’s perceived acceptance–rejection may have on their own psychological development, especially on their personality. 

During early adolescence, the processes involved in the development of personality undergo a transition process until they adjust completely [59]. However, in the case of potentially problematic conflicts, there is relatively little empirical research on the factors occurring during childhood or adolescence that are associated with the differences in the interactions between parents and adolescents [60]. Therefore, more research is necessary in order to account for individual differences. This research should answer questions like “Which parental behaviors may associate with which personality traits in adolescents?” In this regard, the present study focuses on analyzing the relationship between parental acceptance–rejection perceived by adolescents for emotional instability from the early stages of adolescence. Here, special attention will be paid to potential differences between mothers and fathers, as well as to factors like the age and gender of children. Why are we interested in the children’s perception of parental styles? Diverse studies have found a low coincidence between the parent’s and children’s opinions of parental practices [61,62]. The parents have their own perception of their parental practices, sometimes biased by social desirability. Adolescents’ perception has less bias; it is more objective.

## 2. Method

### 2.1. Participants

Initially, 1181 secondary education students were invited to participate in the study. Although all agreed to participate, we excluded 91 cases that presented missing values. Finally, the sample was composed of 1090 students: 49.2% girls and 50.8% boys, whose ages ranged between 11 and 17 years (*M* = 13.97; *SD* = 1.3). The selection of the participants was done through a multi-stage conglomerate sampling and random selection of the groups in the centers that had multiple classes in the first (16.7%), second (25.5%), third (22.9%), and fourth (34.9%) year of Compulsory Secondary Education. The aim of the participant selection was to obtain a representative sample of teenagers. In Spain secondary education is compulsory for teenagers. This fact made the data collection easier in the different education centers. The conglomerate sampling was done randomly selecting 12 centers from a total of 120 Public Compulsory Secondary Education centers of Extremadura. The process consisted in assigning numbers to all the centers and selecting 12 through random numbers generated by a computer. The same process was used to select the classes (between two and four) that were in each first, second, third, and fourth-year course in the 12 centers. Their family situation was as follows: 82.3% of the adolescents lived in a nuclear family with two parents; 11.8% lived with their mother; 1.9% with their father; and 3.4% with other relatives. 

### 2.2. Instruments 

Scale of Affection—Children’s version (EA-H) [61]. This is made up of two factors; each one has 10 items, for which frequency is rated on a 5-point Likert-type scale ranging from “never” to “always”. The first factor, called affection–communication, assesses the children’s perception of the affection, interest, and communication shown by their parents (father/mother) towards them: “He/she comforts me when I’m sad”, “He/she accepts me as I am”, “He/she is affectionate towards me.” The father modality has a Cronbach’s alpha of 0.921, and the mother modality has a Cronbach’s alpha of 0.868. The second factor, called criticism–rejection, evaluates the parents’ criticism, rejection, and the lack of trust (father/mother) toward their children: “Whatever I do seems wrong”, “He/she is discontented when I’m at home”, “He/she would like me to be different”. The father modality has a Cronbach’s alpha of 0.837, and the mother modality has a Cronbach’s alpha of 0.821. The score of each factor ranges between 10 and 50.

Big Five Questionnaire for Children and Adolescents [62] Spanish version [36]. This self-report test measures the big five personality traits (extraversion, neuroticism, agreeableness, openness, and conscientiousness) in a non-adult population. The questionnaire consists of 65 items on a 5-point Likert-type scale (from 1 = “hardly ever” to 5 = “almost always”). For the present study we used the 13 items of the instability factor, which assesses the tendency to discontent and neuroticism, often expressed in mood swings and a tendency to feelings of anxiety, depression, dissatisfaction and irritability: “I get angry easily”, “I am not patient”, “I worry about silly things”, “I get nervous.” It has a Cronbach’s alpha of 0.720.

### 2.3. Procedure 

In order to obtain the data, the questionnaires were administered to each classroom group. First, we contacted the schools to explain the goals of the study and request permission for the questionnaires to be filled out. We followed the ethics code of the American Psychological Association (2010) with regard to parents’ informed consent, as participants were minors. In addition, voluntariness in participation, anonymity, and confidentiality were ensured for all participants, while we made sure that responses and all other data were used for research purposes exclusively. The questionnaires were administered during the school day, usually taking around 20 min—they were filled out in a suitable environment, free from distractions. This study was approved by the Bioethics and Biosafety Committee of the University of Extremadura (No. 0063/2018).

### 2.4. Data Analysis

Prior to the statistical analysis herein, a missing data analysis was performed with the variables included in the models under study. These statistical analyses were made with the SPSS suite (SPSS Inc., Chicago, IL, USA), PC v. 21.0. They consisted of an instrument reliability analysis, a multifactor variance analysis (inter and intra-subject), a linear regression analysis, and lastly, a classification model was created. This model was based on a chi-squared automatic interaction detection (CHAID) decision tree flowchart. 

## 3. Results

First of all, and although this is not a study goal per se, in order to avoid potential confounding factors in the search for the main associations object of study here, we conducted multivariate comparisons of the median scores for emotional instability and for the EA-H factors according to gender, age (early adolescence: 11–13 years; middle adolescence: 14–17 years of age), and the interaction between both variables (Table 1). 

The multivariate analysis (MANOVA) revealed a significant role of gender (Wilks Λ = 0.949, F(5, 929) = 9.958, *p* <0.001, ƞ = 0.051) and the interaction gender–age (Wilks Λ = 0.987, F(1, 1082) = 1.957, *p* = 0.034, ƞ = 0.013), whereas no significant effect was found for age (Wilks Λ = 0.990, F(4, 993) = 2.140, *p* = 0.083, ƞ = 0.013).

As far as emotional instability is concerned, univariate comparisons indicate that girls scored higher than boys, F(1, 933) = 21.563, *p* < 0.001, ƞ = 0.023, as in the case of the interaction gender–age, F(1, 933) = 9.568, *p* = 0.002, ƞ = 0.010. The contrastive analysis of the interaction gender–age showed, on the one hand, that gender differences were only significant among the older participants—girls scored higher than boys, F(1, 1082) = 33.637, *p* < 0.001, ƞ = 0.030. On the other hand, whereas among girls it was the older ones who scored higher, F(1, 1082) = 7.425, *p* = 0.007, ƞ = 0.007), it was the younger ones who scored higher among boys, F(1, 1082) = 6.083, *p* = 0.014, ƞ = 0.030.

With regard to the EA-H factors, the univariate analysis shows that boys scored higher in paternal affect and communication, F(1, 996) = 8.291, *p* = 0.004, ƞ = 0.009) and the younger ones in maternal affect and communication, F(1, 996) = 8.682, *p* = 0.003, ƞ = 0.009). No interaction was found for gender–age (*p* ≤ 0.05). 

In addition, the test for the intra-subject effects indicate that boys and girls perceive higher affect and communication from their mothers, F(1, 996) = 2357.338, *p* < 0.001, ƞ = 0.703, and higher criticism and rejection from their fathers, F(1, 996) = 243.797, *p* < 0.001, ƞ = 0.197. Nevertheless, the interaction with gender shows that the differences for the factor of criticism/rejection are only significant among girls, F(1, 996) = 21.262, *p* < 0.001, ƞ = 0.021, whereas no significant intra-subject effects were found for age or for the interaction gender–age (*p* ≤ 0.05). 

### 3.1. Linear Regression Analysis

Also, in order to corroborate whether children’s perceived affect, interest, communication, criticism, rejection, and lack of trust from their parents (father/mother) are associated with emotional instability, we conducted a regression analysis. By taking into account the results obtained in previous analyses, we designed two regression models for emotional instability according to age (11–13 and 14–17), while keeping gender as a control variable (Table 2).

The lineal regression model for the children aged between 11–13, F(5) = 9.632, *p* < 0.001, R^2^ = 0.170, demonstrates that the factor of maternal criticism and rejection is significantly associated with perceived emotional instability. The lineal regression model for participants aged 14–17 (F(5) = 18.781, *p* < 0.001, R^2^ = 0.194) demonstrates that the factor of paternal criticism and rejection is significantly associated with perceived emotional instability.

### 3.2. Classification Tree

**Figure 1:**
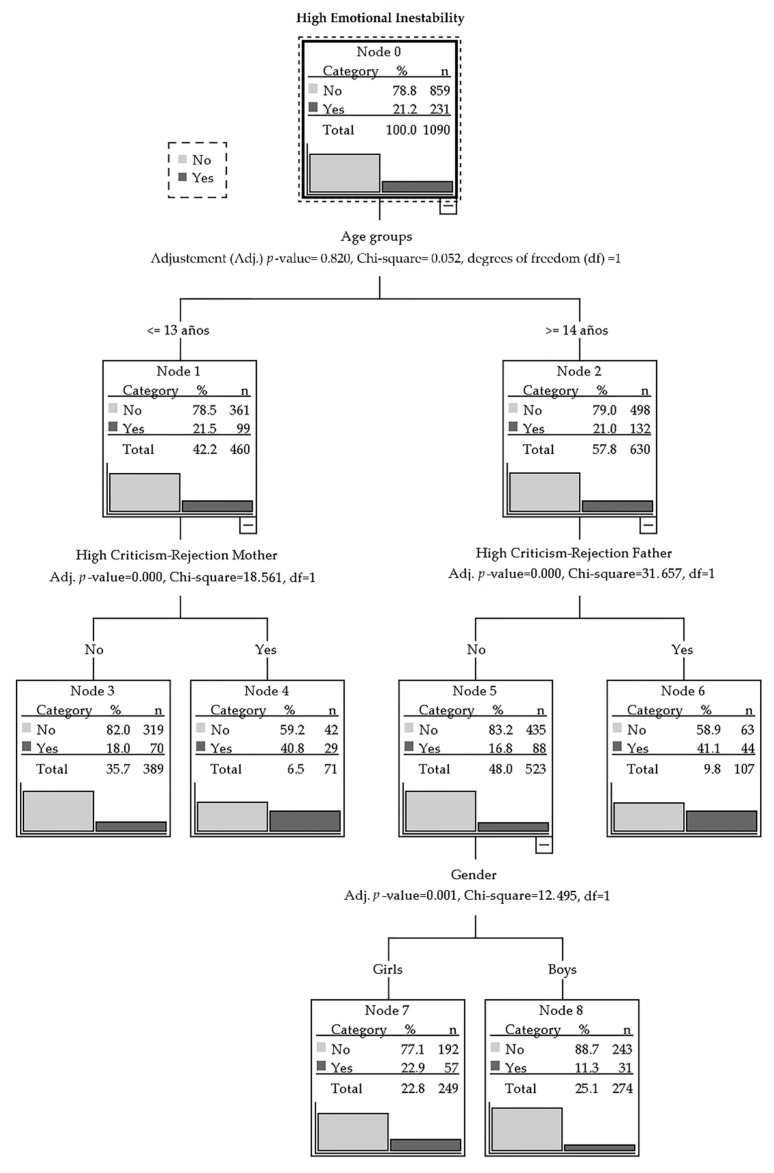
Classification tree for high emotional instability.

Finally, in order to make it possible to interpret the associations we found and find other specific subgroups and relationships that may not be observed through a regression analysis, we made a classification tree (Figure 1). Here, we introduced emotional instability as a dichotomous dependent variable (normal Percentile <80 = 0; unstable Percentile ≥80 = 1), whereas the age groups (force first variable), the four EA-H factors, and gender were used as dichotomous independent variables (maternal/paternal affect and communication; maternal/paternal criticism and rejection; normal Percentile <80 = 0; High Percentile ≥80 = 1). 

This tree correctly classifies 78.8% of subjects (risk = 0.212; SE = 0.012). As we can see in Figure 1, the least likely to present emotional instability (11.3%) are the older boys who do not perceive high parental criticism and rejection (Node 8), whereas the most likely to suffer from emotional instability (Nodes 4 and 6) are both boys and girls—roughly on equal terms—who perceive high criticism and rejection: from their mothers in the case of the younger ones (40.8) and from their fathers in the older age group (41.1).

## 4. Discussion

The present study focuses on the analysis of whether parental acceptance–rejection relates to emotional instability through early and middle adolescence. Our findings show a correlation between children’s and adolescents’ emotional adjustment and family dynamics [14,15]. In general terms, we can see that there exists a clear association between emotional instability with maternal/paternal criticism and rejection. This demonstrates a clear association between children’s upbringing and the development of their personality and socio-emotional adjustment [4]. These results are in line with research that connects parenting styles such as authoritarian or neglectful behaviors, characterized by rejection and lack of communication and/or affection, with internalizing problems such as emotional instability [63,64,65].

The main tenets of the personality sub-theory [58] hold that humans developed the necessity to be attached to emotionally important figures they can rely on [28,29]. These emotional relationships between parents and children are highly associated with of emotional instability in the children [48].

Thus, a question remains: Why we do not find associations between parental acceptance, affection, and communication and emotional instability?

It has been shown that there is an increase in conducts of rejection and hostility toward children during adolescence [57] and that parental rejection is a determining factor, in comparison with acceptance, of the onset of psychological and behavioral problems in minors [42]. More specifically, those who receive rejection from their parents experiment problems associated with certain aspects of emotional instability [36,39,40]. The need for parental acceptance is greater during childhood, later becoming more complex throughout life. Personality experiments a series of changes during adolescence until it eventually adjusts itself [59]. Hence, adults seek the recognition of people they have some emotional attachment to [45].

On a related note, we know that the concept of parental authority and discipline changes through adolescence [66,67]; i.e., in order to keep control, the exercise of power, which is justifiable in childhood, should be replaced by reasoning [68]. Despite this, some parents try to monitor and assess their children’s behavior according to parenting styles of their own, which may have serious repercussions on the socialization of children, especially as they even use rejection as a form of discipline [22,69]. However, given that parent–child interactions constitute a mutual influence [70], one should not overlook the effect of children’s emotional adjustment on the behavior of their parents. Indeed, the effect of the conduct of adolescents on their parents’ behavior has been widely documented [70,71,72,73,74,75]. Emotional instability manifests itself in mood swings, a tendency to anxiety, depression, discontentment, and irritability. As such, it is strongly associated with violence, both verbal and physical [76]. Violent behavior would, in turn, feed maternal and paternal criticism and rejection.

Another question relevant in this discussion is: Why is maternal criticism and rejection in early adolescence and paternal criticism and rejection in middle adolescence associated with emotional instability?

Research on parental acceptance–rejection points toward different results depending on the age of the children [49] and on the gender of parents and children [43,44,45]. We have been able to corroborate these results, at least in part, in this study. However, our results may lead to the conclusion that the changes in family dynamics during the early stages of adolescence result in less maternal rejection and more paternal rejection. Yet, the intra-subject comparisons between maternal and paternal rejection do not yield significant differences for any of the EA-H factors based on the specific stage of adolescence. This would seem to imply that, regardless of the perception of greater maternal/paternal rejection, the maternal rejection is more relevant to emotional instability during the early stages of adolescence, while the same also holds true for paternal rejection at later stages. 

This may have to do with the needs that are connected to developmental changes in personality and in the figures of attachment during the transition from childhood to adolescence. Also, this implies a readjustment in adolescents, their family dynamics, and the roles adopted by mothers and fathers with regard their parental functions. 

However, despite the existing fatherhood culture that promotes the image of a father who is more invested in children’s upbringing from the early stages of life [77,78], boys and girls in our study, in line with the majority of research on the subject, perceive higher affection and communication from their mothers [43,79,80,81,82,83,84,85,86,87,88]. It may be the case that cognitive ability may account for the different consequences of maternal/paternal rejection in the emotional adjustment of children [58,89]. Greater cognitive ability—dependent on maturity—to interpret and discriminate between conducts of acceptance/rejection, as well as to assess the ensuing interactions with their parents, may allow them to handle mother rejection more effectively. 

## 5. Conclusions

The analysis of mental health in children and adolescents has raised great social concern because of its links with disability, suffering, and functional deterioration. This is why the study of risk factors for emotional disorders has become a major research topic worldwide. Indeed, it is at the core of public health policies, representing remarkable expenses for public health systems on a global scale. 

In this regard, the present study makes a significant contribution toward understanding the importance of parental acceptance–rejection in emotional and behavioral problems in adolescents. These are associated with problems both for those who experience them first-hand and for those who are close to them. Thus, this corroborates the notion that the family unit is a key factor in mental health problems [90].

In the face of these problems, intervention and training programs reveal themselves as especially relevant, since they improve families’ knowledge and parenting skills through specific training on non-violent discipline, child development, anger management, or conflict-solving skills [91]. Parental training thus becomes part and parcel of children’s upbringing, as it effectively promotes their development, improves parent–child relationships, teaches conflict-solving skills, and boosts the feeling of satisfaction and competence in parents toward their parenting tasks and responsibilities. 

However, in our opinion, parental training should be oriented towards their own discovery of the fact that their behavior may be the origin of their children’s problems. Training should focus on the importance of providing security and emotional welfare, guiding children’s behavior and their emotional adjustment through childhood, so as to pave the way for emotionally independent adolescents who can face adversity and failure without emotional alterations. Thus, reducing control is one of the most important adjustments that parents must make in order to adapt to the needs of adolescents and stimulate their autonomous development [1]. It is fundamental for parents to be aware that the transition from childhood to early adolescence brings about a series of evolutionary changes in their personality and their attachment figures. This, in turn, involves new needs that are connected to the necessary readjustment in adolescents, family dynamics, or parental roles. 

### Study Limitations and Future Directions

This was a transversal study, and therefore causal associations cannot be made. Also, the exclusive use of self-reports and the use of the children’s perception as the sole collection source of information hinders the possibility of controlling possible respondent bias.

Although children are reliable informants, it is also useful to factor in the point of view of parents. Parent–child relationships are characterized by their bidirectional, interactive nature. Children are not simply passive elements, for their attitude and behavior have a significant impact on parental behavior and this, in turn, influences the development of adolescents. On some occasions, the lack of emotional adjustment may increase parental rejection and criticism, which in turn may intensify children’s emotional reactions. It’s also important to highlight that this is a study that includes only some potential variables associated with the emotional instability of children and their relationships with their parents. This entails the lack of control of social-environmental variables, like the different characteristics from school or the classroom.

Despite these limitations, this study makes interesting contributions to understanding paternal and maternal rejection during the different stages of adolescence. Along these lines, we would like to find out more about whether these differences may be better accounted for by the individual and group effects of paternal and maternal rejection. In the future, we may be able to identify environmental variables that relate to personality disorders that hamper the ability of children to engage in interpersonal relationships. This may be achieved by studying personality in adolescence, parental conduct, upbringing patterns, and/or parenting styles.

## Figures and Tables

**Table 1:** Descriptive data for emotional instability and the factors of the EA-H scale for age and gender groups.

Variables	Gender	Age 11–13		Age 14–17		Total	
		*M*	*SD*	*M*	*SD*	*M*	*SD*
Emotional instability	Girls	33.11	9.38	35.20	8.85	34.25	9.15
	Boys	33.01	9.49	31.09	8.03	31.84	8.67
	Total	33.07	9.42	33.01	8.66	33.04	8.99
Paternal affect–communication	Girls	34.99	11.14	34.91	10.46	34.95	10.77
	Boys	38.23	9.61	36.41	9.94	37.12	9.85
	Total	36.50	10.57	35.72	10.20	36.05	10.36
Paternal criticism–rejection	Girls	18.34	7.29	18.48	7.77	18.42	7.54
	Boys	19.05	7.09	18.77	7.78	18.88	7.52
	Total	18.67	7.20	18.64	7.77	18.65	7.53
Maternal affect–communication	Girls	41.34	7.76	40.35	9.04	40.80	8.48
	Boys	41.43	9.15	39.40	8.46	40.19	8.78
	Total	41.38	8.43	39.84	8.74	40.49	8.64
Maternal criticism–rejection	Girls	17.56	6.48	18.15	7.14	17.88	6.84
	Boys	18.69	7.12	18.75	7.03	18.73	7.06
	Total	18.08	6.80	18.47	7.08	18.31	6.96

*M* = mean, *SD* = standard deviation.

**Table 2:** Factors associated with emotional instability based on linear regression analysis, controlling for gender.

Age Groups	Variables	B	Standard Error	β	*t*	*p*
Age 11–13	Paternal affect and communication	−0.012	0.062	−0.014	−0.201	0.841
	Paternal criticism and rejection	0.154	0.096	0.116	1.609	0.108
	Maternal affect and communication	0.013	0.071	0.012	0.182	0.856
	Maternal criticism and rejection	0.348	0.097	0.243	3.574	0.000
Age 14–17	Paternal affect and communication	−0.072	0.047	−0.084	−1.534	0.126
	Paternal criticism and rejection	0.292	0.076	0.238	3.858	0.000
	Maternal affect and communication	0.104	0.054	0.011	1.744	0.092
	Maternal criticism and rejection	0.079	0.069	0.071	1.145	0.253

B = unstandardized regression coefficient. β = standardized regression coefficient. *t* = obtained *t*-value. *p* = probability. Dependent variable: emotional instability.
